# Clinical Remarks on the Functional Vomiting of Hysteria

**Published:** 1883-06

**Authors:** J. S. Bristowe


### CLINICAL REMARKS ON THE FUNCTIONAL VOMITING OF HYSTERIA. By J. S. BRISTOWE, M.D., F.R.S.

There are few more troublesome affections to deal with than hysterical vomiting, and I suppose that most practitioners of long experience have, at one time or other, been sorely perplexed by cases of the kind. Fortunately, patients seldom die of it, and, even though all kinds of treatment may seem to fail, more or less perfect recovery usually ensues in the long run. I call to mind a few cases which in former years have deeply interested me; especially two, the one, that of a young unmarried woman, a hospital patient, the other that of a lady of mature years, whose hysterical symptoms were induced in the first instance by severe domestic affliction. The younger patient's vomiting dated from a voyage she had made across the Atlantic some time previously to her admission into the hospital. She continued to vomit after everything she swallowed, and came under my care in a state of great debility and emaciation. She remained in the hospital for some time, suffering from what seemed to be extreme irritability of the stomach, which drugs failed to influence, and which was finally benefited, though not cured, by reducing the food administered by the mouth to teaspoonfuls of milk only, and by supplementing these by nutrient enemata. The lady presented many symptoms of aggravated hysteria, besides constant and uncontrollable vomiting coming on immediately after everything that was taken into the stomach. She continued in this state for two or three years, became reduced to the last stage of emaciation and helplessness, and on many occasions appeared to be at the point of death; but she recovered. Some years afterward, having in the interval enjoyed excellent health, she suffered from a recurrence of her malady. In many respects the symptoms were different, but there was a return of the incessant vomiting. She continued in this state for many months, and again she became a living skeleton, and again her life was despaired of. But once more she recovered absolutely; and she remains well. I do not now give these cases in detail, partly because I have said enough about them for my present purpose, but mainly because I hope on some future occasion to devote a special paper to their consideration.

In the spring of last year, another case of aggravated hysterical vomiting came into the hospital under my care. The patient was a distinctly hysterical young girl, who had been constantly vomiting for about four months, and who had consequently become extremely thin and weak. The abdomen was shrunken, but there was no sign of abdominal disease. Nevertheless, she continued to vomit after admission, exactly as she had vomited before, after everything she swallowed, even if it were only a little water. Various remedies were tried without effect; the food was reduced to milk given in diminishing doses, and ultimately in teaspoonfuls, but still she vomited. Raw meat was then administered, but the result was the same. Then for a week or so nutrient enemata were given to the exclusion of all other food; at the end of which time milk was again tried in minute quantities, and again it was rejected as it had been all along.

The question now for the first time presented itself to my mind, "Was it possible that the girl's vomiting was due, not to irritability of the stomach, but to functional affection of the œsophagus, and that she was being slowly starved simply because no food ever reached the stomach?" There was no doubt whatever that she swallowed the food. On this point the ward-sister and all who had to deal with her were unanimous. Indeed, I had myself watched her in the act of taking food; and further, I now made her swallow some milk in my presence. The act of deglutition was—it always had been—perfectly performed, the mouthful descended into the œsophagus, and thus got out of the sphere of voluntary action; and then, at the end of a minute or two, after appearing to suffer from a great deal of discomfort, she brought it up, as was her custom, without violent straining, but with efforts that fairly well resembled those of vomiting.

The reasons which collectively led me to suspect that her food never reached her stomach were partly personal reasons, and partly reasons derived from experience.

The personal reasons were, that there were never any clear symptoms of indigestion; no uneasiness after food; no flatulent distension, or tendency to eructate; and that, so far as I could ascertain, she vomited all kinds of food, liquid or solid, equally, no matter how little or how much was taken. It seemed impossible that she could vomit from the stomach, without the most violent effort, the minute portions of milk, iced water, and raw beef which were often administered to her, which nevertheless she did reject (after swallowing) almost without change and almost without effort.

The reasons derived from experience were mainly furnished by three cases which presented themselves to my mind. A spare, middle-aged clergyman, of nervous temperament, and liable to megrim, has been in the habit for several years past of consulting me about his ailments, of which the most important has been a peculiar spasmodic affection of the œsophagus (so far as I know quite independent of organic disease of the part), which is apt to attack him at the beginning of a meal, and is usually attended by a painful sense of constriction originating in the lower part of the tube, gasping for breath, and faintness. Now and then, also, he brings up during the night a quantity of mucus, which appears to come from the œsophagus. But the special point in his case, which makes me refer to it now, is that some time since he took at night a dose of morphia for the relief of a threatened attack of megrim, without the expected relief, or even sleep, following, until half an hour or so after breakfast next morning, when he became drowsy. He was satisfied that the morphia had lain in his gullet all night, and that it had only been carried into his stomach with his breakfast. His suspicion has since been confirmed; for on several occasions subsequently, his dose of morphia, if it has not been carried on by food taken later, has lain in his œsophagus all night without producing any effect, and either it has been regurgitated in the morning, or its effects have followed his matutinal repast.

The second case was that of a hospital patient of mine, a man over fifty, who had suddenly, about a week before admission, become incapable of swallowing. He had been a healthy man; there was no explanation of his state that I could make out, but he had been wholly without food for a week, and he had consequently become thin, and especially much enfeebled. On making him take food in my presence, I found that he masticated properly, and that the act of deglutition was performed without difficulty, but that immediately what he had taken was violently ejected. The impediment was clearly in the upper part of the œsophagus. Having failed to detect any lesion by external examination, or by looking down the throat, I proceeded to pass a bougie. There was a very slight impediment at the upper part of the œsophagus, which was overcome without difficulty, and the instrument was pushed on into the stomach. The effect was marvellous; the patient swallowed without the slightest difficulty immediately afterward, and swallowed thenceforth as readily as he had always done up to the time of his illness. He came under my care again six months later for a temporary attack of catarrhal jaundice, from which he recovered in the course of a week or ten days. He had had no recurrence of dysphagia.

The third case was one of painful interest to me, for I failed to recognize its nature, and it is mainly, perhaps solely, to this failure, that his death must be attributed. The patient was a young man, whose illness had commenced six months before I took charge of him, and was attributed by him to his having drunk a pot of beer which irritated his gullet as it passed down. From that time he seems to have had constant sickness after food, and to have vomited from five minutes to half an hour after everything he took. He was very thin and weak when I first saw him, but I was unable to detect any evidence of abdominal disease. I attached very little importance, however, to the attributed origin of his illness, and assumed that, as his vomiting was often (indeed, generally) delayed for some considerable time after the ingestion of food, it was due either to pyloric obstruction, or to some functional disturbance of the stomach referable to disease external to it. In other words, though I never ventured to commit myself as to the exact nature of his malady, I believed that he had chronic organic disease; either chronic ulcer or cancer of the stomach, or disseminated cancer or tubercle. There were obvious and strong reasons against each of these views of his case; still, believing as I did that the vomited matters came from the stomach, I did not see my way to any other explanation, and I never thought of passing a bougie or of feeding him by the œsophagus tube. His vomiting was persistent up to the time of his death. At the post-mortem examination, his stomach and other abdominal organs were all found to be healthy, and the only lesion discovered was dilatation of the œsophagus, with hypertrophy of its walls. I now naturally attached more importance than I had done to the history which he gave of his illness; I admitted that his dilated and flaccid œsophagus had formed a virtual impediment to the entrance of food into the stomach; I became impressed with the important practical fact that in œsophageal obstruction vomiting may be delayed for half an hour or more, as it is habitually in pyloric stricture; and, above all things, my unfortunate experience taught me the importance, in all obscure cases of persistent vomiting, of not omitting to examine the œsophagus, or to try the effects of injecting food directly into the stomach. The following are the details of this case:

*Dilatation of the Œsophagus with Persistent Vomiting—Death.*—J. B., a gardener's laborer, æt. twenty-four, was admitted into St. Thomas's, under my care, on June 7, 1879.

He was perfectly well (he said) until six months previously, when one day he drank about a pint of beer, which had a bad taste, caused some irritation along the gullet, and made him sick. The next day he had pain and difficulty in swallowing, and vomited after every meal. The vomiting had continued ever since, coming on from five minutes to half an hour or even an hour after ingestion, and induced not only by food, but by iced water and by medicine. He thought, however, that solids were less provocative of sickness than fluids. He had at no time, since the very first, had pain or difficulty in the act of deglutition, but had often suffered more or less pain behind the sternum and extending to the umbilicus, which came on after food and was relieved by vomiting. He had never vomited blood. He had generally had a desire for food, and had suffered only slightly from thirst. The bowels had been much confined. He had had a slight cough on and off for some time, and said that his sputa had occasionally been streaked with blood. He had lost flesh and strength latterly.

He was a thin, delicate-looking man, and patches of dilated vessels in his cheeks added to the unhealthiness of his aspect. His tongue was clean and moist, but rough; his appetite was fair; his bowels constipated. He complained of a slight dry cough; but on physical examination there was no evidence of disease either of the lungs or of the heart. The respirations were 18; the pulse 120, feeble, small, and regular. The abdomen was soft and flat, and no tumor nor enlargement of any organ nor tenderness was discovered in it. The urine was small in quantity, free from albumen, and its specific gravity was 1028. The temperature was subnormal. There was no œdema of the limbs, and no enlarged glands in any accessible region. The vomit consisted mainly of matters which had been swallowed, and presented no pathological products under the microscope. The motions were solid and of healthy character. He was treated with bismuth and put on milk diet.

It would be tedious, nor would it be instructive, to reproduce the periodical notes that were taken of the patient's case from the time of his admission up to August 25, the day of his death; for, beyond the fact that there were progressive asthenia and emaciation, the symptoms varied but little from week to week.

He was treated dietetically mainly, by milk, and latterly by wine in addition, given in small quantities by the mouth at frequent intervals, and by nutrient enemata administered from two to four times a day. This treatment had the effect apparently of diminishing his sickness from time to time, and even of arresting it occasionally for a day or two; but on the whole the vomiting continued generally after everything he took, and from five minutes to half an hour or so afterward, and at times was severe. The vomit was generally merely what he had swallowed mixed with mucus, but occasionally it was dark and offensive, and had an unpleasant taste. Streaks of blood were observed in it from time to time. He generally complained of pain behind the sternum after swallowing, and occasionally also of pain at the episternal notch, a few inches below the left nipple, or at the umbilicus. It was noted on one or two occasions that the vomit came up without any straining. And, during the earlier part of his residence in hospital, he manifested a desire for food. He complained but little of thirst. His tongue was generally coated and sometimes dry.

The abdomen never became tumid. On the contrary, it got more and more hollow, and was always free from tenderness and evidence of tumors. Shortly before his death, it was remarked that what appeared to be a narrow, thick-walled tube could be felt extending transversely across the abdomen above the umbilicus, and could be freely moved upward and downward. It was assumed to be the contracted stomach.

The bowels were for the most part confined, but during the latter part of July the patent suffered from diarrhœa.

The daily yield of urine varied from 10 to 18 ounces. Its specific gravity was high, but it was free from albumen and other abnormal matters. At one time he complained of pain and difficulty in passing it.

He suffered more or less from cough during the whole of the time he was under observation, and at times it was very troublesome, and attended with mucous expectoration, which was occasionally streaked with blood. There was never any clear evidence of pulmonary phthisis, but respiration was harsher and the voice resonance louder at the right than at the left apex, and some variable crepitation and rhonchus were observed here and there.

The heart's action was very feeble, and the pulse, which was always small and regular, sank until latterly it was only 52 in the minute.

His temperature never rose above 98.7°, and was almost invariably subnormal. It tended also to sink from the time of his admission to the day of his death. During July it ranged for the most part between 97° and 94° in the axilla. In August it sank still lower, and before his death fell to 92.8°. Latterly, also, he complained much of cold, and his hands and feet became cold and livid; occasionally he perspired. There was a uniform loss of body-weight from first to last. He weighed 8 stone 3 pounds when he first came into the hospital. He weighed only 5 stone 9 pounds on August 16. He was often very low-spirited during his illness, and was apt to cry; and shortly before his death he was occasionally delirious. He was conscious, however, to the last.

*Autopsy.*—The œsophagus was much dilated throughout its entire length, and full of fluid. It measured five inches in circumference at its upper part, and three inches just above the cardia. There was no stricture. The muscular coat was hypertrophied. The mucous membrane was thickened, generally pale, but presenting a few injected vessels, and thickly studded throughout its whole length with shallow circular pits, which appeared to correspond to dilated mucous follicles.

The stomach was much contracted, and its mucous membrane presented a few patches of congestion. The intestines were contracted and healthy. All the other abdominal viscera and the peritoneum were free from disease.

The lungs were congested, and the base of the right one was collapsed; otherwise they were healthy. Heart healthy.

The lessons applicable to my hysterical patient, which these three cases taught, were that vomiting from the œsophagus might simulate vomiting from the stomach; that œsophageal spasm might form a persistent obstacle to the passage of food; and that small quantities of alimentary or other matters might fail to excite the proper peristaltic movements of even the healthy gullet, and that hence the feeding of patients with teaspoonfuls of food, as is done in cases of irrritable stomach, might fail both to impart nourishment and to throw any light on the condition of the patient's stomach.

The results of my experiment will appear in the narrative which I now proceed to give.

*Hysterical Vomiting.** *Cure.*—A delicate-looking girl, fourteen years of age was received into one of my beds in St. Thomas's on September 21, 1881. She was suffering, we were told, from an hysterical affection of the right hip, which first showed itself in July, 1879, and which had continued without cessation, but with varying severity, up to the date of her admission. It did not appear that she had ever before suffered from serious illness; she had never had fits; she had never been hysterical (in the popular sense of the term); the catamenia had not appeared.

At the time mentioned she fell into a state of languor and weakness, and the right hip became very painful—the pain running down the front of the thigh, and extending into the knee. The pain increased in severity until October, at which time she limped in walking, and only put her toes to the ground. This condition

* An almost identical case of œsophageal spasm, only in a young boy, is recorded by Mr. Hulke. Clinical Soc. Trans., Vol. VI.

continued without change to the end of the year, when some improvement took place, which was maintained during the greater part of 1880. In the autumn of that year her symptoms became much aggravated; she seems to have had considerable lumbar pain, and is said to have had sciatica. She then took to her bed, and never left it till July, 1881. There was no improvement, however, after this time, and she was at about her worst when I first saw her.

Then she complained of some pain in the back, but mainly she suffered with her hip. The joint was slightly flexed, and when she attempted to walk (which she did unwillingly, and only with assistance) the right lower limb was kept bent at the hip and knee joints, and she limped, but she planted her foot flat upon the ground. The joint was excessively tender, especially behind the greater trochanter, but there was no swelling, redness, or increase of temperature, and it was distinctly observed that she complained no more when the joint-surfaces were pressed against one another, or when the ligaments were stretched, than she did when the skin was simply touched. There was some rigidity about the joint, but no wasting of muscles. There was nowhere any loss of sensation or of the tendon reflexes, but the superficial reflexes were feeble. The affection had been regarded as hysterical, prior to admission, and in this opinion I and others who saw her in the hospital concurred. While under treatment, she manifested a tendency to sob at times, and the condition of her hip varied a good deal, but she left apparently much improved, on December 10.

She was re-admitted on May 15, 1882. It appeared that, soon after she left the hospital, she began to vomit after food, and before long, after everything she took, the sickness coming on immediately; that she rapidly lost flesh and strength; and that, although the hip-affection remained, it formed a less prominent subject of complaint than it had done previously.

She was much emaciated (weighing only 3 stone 3½ pounds), very feeble, and confined to bed; her cheeks were a little flushed, and her face (which rarely varied) wore a mixed expression of apathy and martyr-like resignation; her skin was dry; her pulse feeble and slow; her temperature normal. She vomited after everything she took; her bowels were constipated; her urinary secretion was normal; she had no abdominal pain. The belly was hollow, and presented neither tenderness nor lump. The catamenia had not appeared. Her hip was tender, and the joint was kept partly flexed, but she complained of it much less than when she was in the hospital last.

From the first she continued to vomit after whatever was taken; the vomit consisting mainly of the food swallowed and mucus, and the sickness generally coming on a few minutes after ingestion. It was sometimes, however, delayed for ten minutes or a quarter of an hour. It appeared, nevertheless, that a small proportion of her food was retained. After a day or two's experience, she was ordered to take a dessertspoonful of milk only every half-hour, which she vomited. The quantity was then reduced to a teaspoonful, which she likewise vomited; but at the same time nutrient enemata were directed to be administered twice daily. As the vomiting continued without abatement after every kind of fluid swallowed, no matter what its bulk, it was determined to make a trial of small quantities of solid food, frequently given. Pounded raw beef, mixed with currant jelly, was selected, of which she took a teaspoonful at a time. This, however, returned as everything else had returned. A fortnight after admission (the rejection of food continuing unabated) it was determined for a few days to give the stomach entire rest, and to feed her solely with nutrient enemata, of which at first three, and subsequently five, were given daily. She was treated thus for about ten days, and during this time vomited only after taking medicine, which was consequently discontinued after a day or two. During the latter period no particular change was observed in her condition; she presented the same manner and appearance as on admission; she complained of no pain excepting in her back and right hip; she was generally very restless and sleepless at night; she had no desire for food or drink; she did not feel sick; her bowels were confined; her temperature was generally subnormal, as it had been nearly ever since admission, and often sank to 96°; her pulse, which was extremely feeble, ranged between 42 and 60. She had lost only one pound in weight in a little more than three weeks.

At the end of this time the administration of milk in teaspoonful doses was recommenced—at first only three or four times a day, and then every hour. But again we were disappointed, for after every dose of milk, milk was speedily vomited. In fact, her stomach appeared to be just as irritable now as it had been at first. After a day or two, a grain of opium in the form of a pill was given two or three times a day; but this treatment had no effect. The administration of milk by the mouth was persisted in for four or five days, at the end of which time, feeling a good deal puzzled and disheartened, I gave more serious thought to the incidents of her case than I had previously done, and discussed them fully with my class. I had hitherto assumed that she was suffering from extreme irritability of the stomach, and that it was this irritability which caused her to vomit constantly. But I now called to mind that she had never complained of actual pain or tenderness in the region of the stomach; that she was not flatulent; that her vomiting was an easy process with her; and especially that she brought back the greater part of the minutest quantities of food taken, and in whatever form it was taken. And I asked myself the question, "Was it possible that the bulk of her food never entered the stomach at all, but was retained in the œsophagus and thence regurgitated?" I had other reasons, which I have already fully explained, which helped to suggest this question, and inclined me to answer it in the affirmative. I now made the girl swallow a dessertspoonful of milk in my presence, and watched the progress of events. She swallowed it without difficulty, and it evidently went beyond the influence of the pharynx; then she appeared to suffer from some discomfort, and in the course of a minute or so, without any very violent effort, but with a certain amount of spasmodic action, the milk was gulped up into the mouth.

I then (on June 11) got Mr. Pitts, the resident assistant surgeon, to see the patient with me. And at my request, and in my presence, he passed a medium-sized India-rubber tube along the œsophagus into the stomach, and then injected into that organ about three ounces of milk. There was a little impediment to the passage of the instrument in the lower part of the tube; but it was readily overcome, and evidently was not due to any organic disease. The milk thus injected did not cause any feeling of sickness, and remained in the stomach without causing discomfort. She did, immediately after the removal of the tube, regurgitate a small quantity of milk, but this was clearly only the milk which escaped into the œsophagus during the withdrawal of the instrument.

It was intended to feed her daily by the tube, but she never required it again during her stay in the hospital. For the next day or two she took milk in small quantities, returning a little of it only occasionally. Two days after the use of the tube, she began to take a tablespoonful of milk every hour, which she retained. The next day a teacupful of milk, with a little tea in it to give it flavor, was ordered to be given several times a day instead, and a little bread and butter was added. These also were retained. The next day her allowance of food was increased by a small quantity of custard-pudding; and thus by degrees her diet was improved in quality and increased in quantity, until, at the end of two or three weeks (or about June 28), she was taking daily a fair quantity of milk, together with two eggs, fish, pudding, and bread and butter. The nutrient enemata, however, were persisted in for a day or two longer, and were then discontinued, partly because their more nutritive ingredients had been withdrawn for administration by the mouth, partly because the bowels, which had hitherto been constipated, became loose. The diarrhœa troubled her for about a fortnight, and had to be treated by astringents. But she continued to take food in increasing quantities up to the time of her leaving the hospital.

It must be observed that the patient to the last appeared to have no desire for food, and to derive no pleasure or comfort from taking it. She took it only because she was compelled; but she took it without difficulty or discomfort. Only on one or two occasions did she vomit any of it. She did not gain flesh very appreciably, and in fact, when she left only weighed two pounds more than she had done on admission, and three more than at her period of greatest enfeeblement and emaciation. The continued diarrhœa may, to some extent, have retarded her progress in this particular, but in other respects the improvement, if slow, was marked; she certainly grew stronger and more cheerful, and her aspect and complexion assumed the characters of health. Moreover, her temperature, which for the greater part of her stay in the hospital had ranged between 95° and 98°, during the last few weeks seldom fell below 97°, and generally varied between one or two tenths of a degree above 98° and one or two tenths below it. The pulse was generally very slow throughout her illness, varying perhaps between 40 and 60, but latterly it rose occasionally to 70 or 80.

She left the hospital on July 29, cured of the vomiting, and generally benefited in health, but not so much benefited as I could have wished. The fact is, she fretted so much, and so persistently, to go home, that at length, fearing her constant fretting might be retarding her convalescence, I reluctantly complied with her wish. It was clear, however, that though she was thus cured of one important outcome of her hysterical condition, the fundamental malady still remained. The hip-joint continued painful, and I was scarcely surprised to hear that, a month or two later, during my absence from town, there had been a recurrence of the vomiting, and that her mother had brought her to the hospital to have the œsophagus tube re-introduced.

I have little to add by way of comment. There is no doubt, of course, that in most cases of hysterical vomiting, it is the stomach that rejects the food. But it is obvious that in an undetermined minority of cases of such vomiting, of which my case is an example, it is the œsophagus rather than the stomach that is in fault, and if, in such cases, the irritability or spasm of the gullet can only be overcome, and the food swallowed be allowed to reach its destination, the vomiting will cease. If one has reason to suspect the latter condition to be the cause of the patient's symptoms, it is fortunately easy to put the question beyond doubt by having recourse to the œsophagus-tube or stomach-pump ; and, if the answer be in the affirmative, to cure the patient of her malady by the repeated use of the instrument and artificial feeding. There is reason, however, to hope that a single introduction may suffice to effect a more or less permanent cure.

How often one has reason to wish that the past, with its misapprehended experience, could be recalled ! I have often thought, since I learnt the lesson which my hysterical girl taught me, that the two cases which I quoted at the beginning of my paper were cases of the same kind as hers, and might have been cured with comparative ease and rapidity.—*Practitioner.*

